# Metabolic Syndrome Detection Based on Classification of Electrocardiography Signals

**DOI:** 10.3390/s25216752

**Published:** 2025-11-04

**Authors:** Edilaine Gonçalves Costa de Faria, Euler de Vilhena Garcia, Cristiano Jacques Miosso

**Affiliations:** 1 Electrical Engineering Graduate Program, University of Brasilia (ENE/UnB), Brasília 70910-900, Brazil; edilaine@ieee.org; 2 Biomedical Engineering Graduate Program, University of Brasilia at Gama (PPGEB/UnB), Brasília 72444-240, Brazil; miosso@ieee.org

**Keywords:** support vector machines (SVMs), RobustBoost, convolutional neural network (CNN), metabolic syndrome, cardiac axis

## Abstract

Metabolic syndrome (MS) components, mainly correlated with insulin resistance and diabetes, constitute physiological disturbances that are objectively detectable based on physiological and anatomical measurements. In particular, the scientific literature indicates clear associations between features extracted from electrocardiograph (ECG) signals and MS. However, there exist few scientific studies related to MS detection by means of ECG signals, specially in automatic computer aided systems. This paper aims at developing and evaluating automatic tools for possible MS detection based on ECG signals. To evaluate how accurately and precisely the developed classifier systems detect MS from ECG signals, we use the following procedures. Initially, we use algorithms that automatically extract Q, R, and S peaks from ECG waveforms. Subsequently, we extract temporal features mainly associated with averages and variances of intervals and ratios between successive Q, R, and S peaks. We also use features describing the cardiac axis. The features are then used for training and testing classifier systems, including Support Vector Machines (SVMs) and RobustBoost classifiers. We also test the use of classifiers operating on raw ECG signals, without preliminary explicit feature extraction. The tested models constitute different configurations of Convolutional Neural Networks (CNNs). Our results indicate that it is possible to classify ECG signals in two different classes, separating people with MS from a control group, with statistically significant results. SVM, RobustBoost, and CNN models obtained average accuracy values equal to 94%, 89%, and 98%, respectively. These results indicate that automatic computer-aided diagnositcs of MS can be added to standard ECG clinical exams.

## 1. Introduction

The diagnosis of metabolic syndrome (MS) builds upon a set of clinical signs related to insulin resistance, such as hypertension, higher blood glucose, increased abdominal obesity, and decreased high-density lipoprotein (HDL) blood level. These factors cause alterations in the metabolism and, consequently, changes in the cardiovascular system [1,2,3].

Although there is no consensus regarding the quantitative criteria for MS determination, the scientific literature shows that (i) the usual signs of MS are related to insulin resistance and to diabetes; (ii) pre-diabetes patients with MS may develop diabetes later; and (iii) people with MS may also have cardiovascular diseases [4,5,6,7,8].

In this research, the markers for MS diagnosis are related to criteria by the National Cholesterol Education Program’s Adult Treatment Panel III (NCEP-ATP III), which states that the person is diagnosed with MS when he/she has at least three components according to the levels presented in Table 1 [3].

According to the Institute for Health Metrics and Evaluation (IHME), from 1990 to 2018, death figures from diabetes have evolved from 5th to 3rd place in the rank of death causes in Brazil. Diabetes-related deaths are associated with high levels of fasting blood glucose and increased body mass index. These factors are also responsible for increased risks of cardiovascular diseases [9].

The modifications in the features extracted from ECG signals can be explained by physiological and pathological changes associated with metabolic syndrome (MS). The literature presents studies indicating that patients with MS exhibit cardiovascular dysfunctions, which are reflected in alterations in the QRS waves, the cardiac axis, and other ECG parameters.

MS risk factors may be associated with heart failure and sudden cardiac death [10,11]. Also, current evidence supports the link between MS and cardiac disorders by heart rate variability analysis [12,13,14,15]. Rate variability in MS has been reported both in long-term studies [16] and in short-term ones [17]. However, we did not find many scientific articles mentioning approaches for detecting MS using only ECG signals.

The basic purpose of an automatic pathology detection system based on ECG system analysis is to obtain clinically relevant information in order to separate ECG in two categories—healthy or pathological. Some initiatives are based on ECG feature extraction (e.g., ECG intervals and segments, QRS and T wave axes, and morphological analyses of ECG waves). Other studies include deep learning techniques, such as those using Convolution Neural Networks (CNNs), in order to perform ECG classification, e.g., for the detection of cardiovascular diseases. In this type of classification, it is not necessary to develop a separate preliminary algorithm for previously extracting features of ECGs. Rather, the feature extraction is performed at the first network layers using linear filters that are adjusted at the training stage and nonlinear operations that are previously selected when defining the models [18,19,20,21,22]. We emphasize that CNNs have been successfully used to classify and analyze time domain signals in several applications [23,24,25].

In this paper, we compare the performance of three different types of classifiers of MS detection based on ECG signals, with and without explicit preliminary features extraction: Support Vector Machines (SVMs), RobustBoost classifiers, and Convolutional Neural Networks (CNNs).

## 2. Methods

We start by describing the methods we implemented for extracting ECG features in Section 2.1. Next, we present the ECG datasets used in Section 2.2. The classification approaches themselves are presented in detail in Section 2.3.

### 2.1. Algorithms for Classifying Electrocardiogram Signals

#### 2.1.1. Features Extraction of Electrocardiogram Signals

In our first proposed ECG classification approach, we started by detecting QRS complexes. We used the Pan–Tompkins algorithm [26] in an implementation developed at the Research Institute of Rochester General Hospital, Rochester, New York, United States [27]. This algorithm starts by detecting the R peaks of the ECG signal. Then, it extracts the Q and S peaks by estimating the first-order ECG derivative and then searching for sign inversions in this derivative. In order to find the S peaks, we searched for the local minima positions by using the approach of searching derivative inversions. We selected the minima locations that were closest to the R peaks and to the left of such peaks.

**Figure 1:**
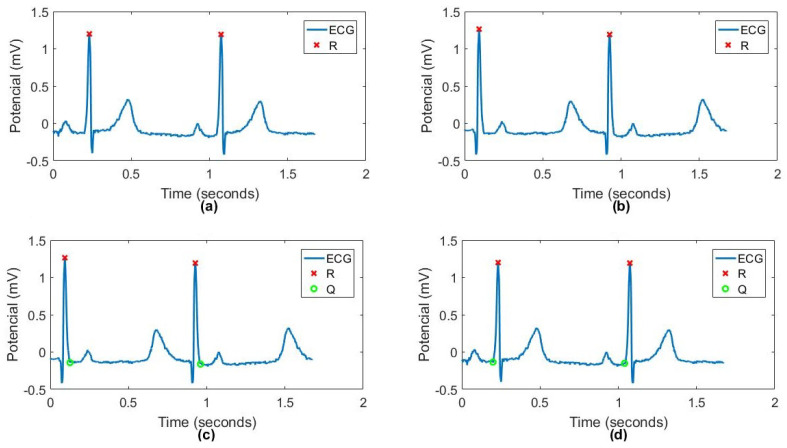
Example of a four-stage algorithm for Q peak detection. (**a**) Stage 1—Original ECG Signal with detected R peaks; (**b**) Stage 2—Mirrod ECG Signal, Stage 1 signal mirrod; (**c**) Stage 3—Mirrod ECG Signal with detected Q peaks; (**d**) Stage 4—Original ECG Signal with detected Q peaks, Stage 3 mirrod.

Regarding the detection of Q peaks, we used a Pan–Tompkins variation that improved the detection accuracy in our tests, as determined by visual inspection of a subset of random signals in the database. We started by time-flipping each signal according to the relation

(1) $$x_{i}\left[n\right]=x\left[N-n\right]\;\;\forall \;n\in \left\{0,1,\ldots ,N-1\right\},$$

where *x* is the original signal, $x_{i}$ is the time-flipped signal, and *N* is the signal length. Figure 1 shows an original signal and its time-flipped version. After time flipping, we repeated the S-peak detection approach in order to extract the Q peaks.

Based on this detection, we extracted time domain features, such as averages and variances of specific types of intervals, as well as ratios and axes between the Q, R, and S peaks. We used, for this feature extraction, leads I and aVF, which are orthogonal leads from the frontal plane and are normally used in studies that involve the cardiac axis of ECG signals [28,29]. Furthermore, the determination of the cardiac axis is a composite metric that involves the evaluation of different leads, which implicitly implies a feature selection by aggregation.

Table 2 details the features we extracted from ECG signals using leads I and aVF.

For the machine learning methods, the strength of association between the occurrence of metabolic syndrome and each ECG feature in every epoch (basal, +30 min, +60 min, +90 min, +120 min) was evaluated by Pearson correlation. Values were considered significant if *p* value was below 0.05. Additional information about the influence of individual features on classification was assessed by Principal Component Analysis (PCA).

PCA creates orthogonal vectors composed of linear combinations of all 30 ECG features and sorts the correspondent eigenvalues. The highest eigenvalues are associated with most of the variability in the original data so that we can reduce data dimensionality by retaining only the associated eigenvectors with the highest eigenvalues. As regards specific feature relevance, we searched for low-value coefficients among the linear combinations in each retained eigenvector. All coefficients with values below 0.2 were considered irrelevant in the vector composition, and the remaining ECG features were compared to the ECG features table of correlations (Section 3.3) for agreement.

#### 2.1.2. Validation of the Used Algorithm for ECG Peaks Detection

In order to validate the peak detection algorithm used, we used Bland–Altman plots to compare the modified Pan–Tompkins algorithm with a manual detection procedure over an ECG database that is appropriate for arrhythmia detectors research [30,31]. The Bland–Altman plots allowed us to evaluate whether there were significant differences in the two extraction procedures and whether they were therefore expected to change the diagnosis results [32].

### 2.2. The ECG Databases

To test the detection peaks algorithm, we used a database of ECG signals from Physionet named *The noise stress test database*. The dataset includes 30-min ECG signals from derivation V1. The signal-to-noise ratios (SNRs) correspond to 24 dB, and the sampling frequency equals 360 Hz [30,31].

In addition to this dataset, we used a set of ECG signals from a research that associated alterations in ECG signals with to insulin resistance in MS individuals. This study was performed by an Applied Biophysics and Bioengineering Group (GBBA, from the Spanish description *Grupo de Bioingeniería y Biofísic Aplicada*) [33]. This database initially appears in [13].

The participants of this research are from two distinct groups, which include, respectively, 15 individuals with MS and 10 from a control group. All subjects are males, with ages between 20 and 44 years, without known cardiovascular diseases. The subjects were reportedly nonsmokers and reportedly did not use illegal drugs. In the MS group, the subjects presented obesity, mainly of the abdominal type [13]. No other information related to individual characteristics of these participants was found. For example, there was no individual information regarding all specific MS markers for each individual. Therefore, in our research, we did not evaluate the influence of each MS component over the ECG signal.

These data were obtained during the Oral Glucose Tolerance Test (OGTT). This is a test in which the individuals need to be fasted at the beginning (then, blood tests are performed before and after the ingestion of 75 g of liquid glucose). This test was performed in 5 different stages. The first was the fasting stage, which corresponds to a basal state. The others were acquired after glucose intake at intervals of 30 min—30, 60, 90, and 120 min after glucose ingestion. In addition, the GBBA research also measured ECG signals during these different stages [13].

This database has 12-lead ECG signals that were acquired for 15 min using Cardiosoft v6.7, with a sampling frequency of 1 kHz and a resolution of 16 bits per sample [13,34].

### 2.3. Classification Methodologies

#### 2.3.1. Development of Classifier System

In building MS detection methods, we compared results from different classifiers, such as Support Vector Machines (SVMs), Ensembles (specifically, RobustBoost classifiers), and Convolutional Neural Networks (CNNs).

We started with the SVM and RobustBoost approaches. These two classifiers are typically trained using supervised learning and typically operate on features that were explicitly extracted beforehand, instead of operating over raw signals. The SVM is well discussed and analyzed in the scientific literature [35,36,37]. The RobustBoost classifier is also well known and is a type of ensemble [38]; it has several important applications in biomedical fields [39,40].

Regarding CNNs on the other hand, they typically receive complete, raw signals or images [41,42], and during training, they learn to extract the relevant features extraction automatically. The literature presents CNNs applied to ECG signals, including the development of online-mode monitoring systems [43]. In this research, 12-lead ECG signals were used to train the CNN.

#### 2.3.2. Data Separation for Classifiers Training and Testing

During the classifiers’ training stages, the data were divided in two parts: one for training and another for testing. We used the *k*-fold cross-validation, a type of cross-validation used for accurate performance estimation [44,45].

**Figure 2:**
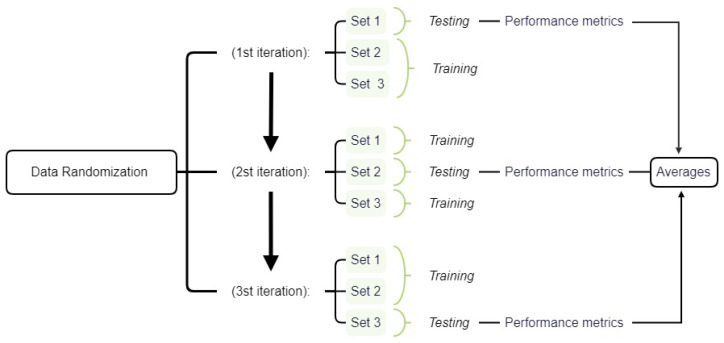
Flowchart of the stages of training and testing of classifiers in an example of k-fold, with k equal to 3.

In *k*-fold, we first randomly divide the training and testing data into *k* sets. One of these groups is reserved for testing, while the other k−1 groups are used for training [44,45]. All relevant performance metrics, such as accuracy, precision, sensitivity, specificity, and F1 score, are then obtained based on applying the training classifiers to the test set. The process is repeated to a total of *k* times using a different test group each time [44,45]. At the end, the performance metrics estimates correspond to the averages of the corresponding metrics obtained for each fold. Figure 2 illustrates this general procedure by showing the classification stages for k=3.

To mitigate the risk of overfitting, we used 10-fold cross-validation and monitored performance metrics. In this study, we used k=10, which we identified as a common minimum value used in this type of application [45].

#### 2.3.3. Classification Algorithms Parameters

**Figure 3:**
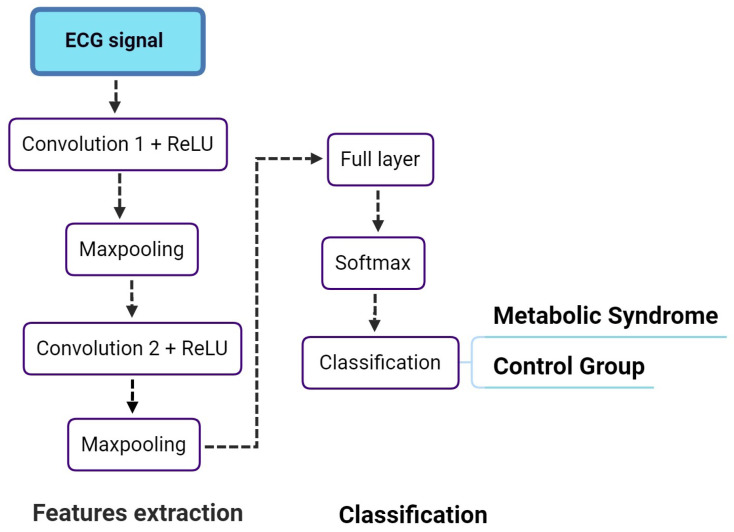
Convolutional Neural Network layers used for classifying electrocardiography signals as belonging to the control group or to the metabolic syndrome group.

**SVMs.** For SVMs training and testing, we used the Radial Basis Function (RBF), which is the function that is most commonly used as the SVM kernel—corresponding to the inner product between the transformed representations of two separate vectors of features [46].
**RobustBoost Classifiers.** Regarding the RobustBoost classifiers, we used 100 learning cycles and tree-based basic classification models. These parameters were defined in preliminary tests in which we detected that fewer than around 100 cycles lead to lower accuracies, and more than this value did not lead to further detected improvement.
**Convolutional Neural Networks.** In the case of CNNs, we noted a greater challenge regarding training the models based on the original, unprocessed database examples. To deal with this challenge, we divided each signal into 300 time domain windows, leading to a larger number of training examples, even though there were several segments for each of the subjects. In this context, we separated examples for testing belonging to subjects that were not used in the training and validation stages (i.e., all time domain signals used for testing corresponded to subjects not having any segment used for training or validation). The CNN models was designed with 10 layers divided into two main stages, namely, the features extraction and the classification stages. Figure 3 show these stages.

In the features extraction stage, the first layer receives the raw ECG signal. CNNs extract information from the entire signal, so the information from the P and T waves may already be included in the set of features extracted by the networks. Research examples present a detector for changes in the ECG, such as atrial fibrillation and arrhythmia, using deep learning [47,48,49]. Furthermore, in this work, we address two critical aspects: analyzing the maximum possible signal (as done by the CNN) and adding metrics related to the cardiac axis and QRS complex morphologies. Thus, this approach, which combines clinical and computational strategies, reduces the need for large amounts of data for training, as seen in purely computational approaches.

Following features extraction, there are the following layers: a two-dimensional convolutional layer with 100 filters and size 5×5; one Rectified Linear Unit (ReLU) layer; and a two-dimensional maxpooling. Next, the network includes a second two-dimensional convolutional stage with 100 filters with size 8×8, followed by a ReLU layer and a two-dimensional maxpooling. The classification layer has one two-dimensional full layer, a softmax layer, and a classification output layer. These layers parameters were chosen during empirical tests until training and validation epochs suggested no important overfitting or underfitting.

Also, we defined the following configurations, which lead to the best comparative statistical results in our experiments: stochastic gradient descent with impulse for the training algorithm; initial learning rate of 0.0001; initial learning rate multiplied by 0.1 at every 8 epochs; L2 regularization factor of 0.0004 (reducing overfitting); maximum number of epochs of 5; mini batch size for each iteration of 30.

#### 2.3.4. Performance Metrics

To evaluate the classifiers, we applied statistical analyses to the results of the tests stages. These statistical analyses were performed by using the confusion matrix, which includes the numbers of true positive (TP), true negative (TN), false positive (FP), and false negative (FN) values. Based on the confusion matrix, we then computed the false positive rate (FPR), the false negative rate (FNR), the positive predictive value (PPV), the negative predictive value (NPV), the sensitivity ($S_{e}$), the specificity ($S_{p}$), and the accuracy ($A_{\mathrm{cc}}$). In computing these statistical metrics, we used the standard definitions [50,51], that is,

(2) $$\mathrm{FPR}=\frac{FP}{FP+TN},$$

(3) $$\mathrm{FNR}=\frac{FN}{TP+FN},$$

(4) $$\mathrm{PPV}=\frac{TP}{TP+FP},$$

(5) $$\mathrm{NPV}=\frac{TN}{TN+FN},$$

(6) $$S_{e}=\frac{TP}{TP+FN},$$

(7) $$S_{p}=\frac{TN}{TN+FP},$$

and

(8) $$A_{\mathrm{cc}}=\frac{TP+TN}{TP+TN+FP+FN}.$$

### 2.4. Statistical Hypothesis Tests

We performed statistical hypothesis tests with a 5% confidence level. First, we analyzed whether the data followed a normal distribution by using the Kolmogorov–Smirnov (KS) normality test.

This study consists of a case of independent samples. Furthermore, the KS test indicated that the features did not follow a Gaussian distribution. Therefore, following the KS test, we used a nonparametric statistical test (the Mann–Whitney) for the following evaluations.

## 3. Results and Discussion

In our experimental tests, we evaluated the use of different ML approaches for detecting MS markers based on ECG patterns. Some methods attained up to 99% in terms of accuracy, precision, sensitivity, and other metrics, as we detail in this section. However, some aspects influenced the final performance and must be properly addressed.

In this section, we detail the observed aspects regarding the algorithms’ performance and robustness.

### 3.1. Peak Detection Validation

The experimental tests in which we applied the Bland–Altman method over *The noise stress test database* suggest that, after the Q, R, and S peaks detection, the diagnosis could be affected by noise associated with movement artifacts. As a result of these artifacts, the algorithm’s performance was reduced, as discussed below.

Table 3 shows a summary of performance results classifiers. The accuracy value up to 99.3% indicates that it is possible to detect individuals with MS or health using just ECG signals. The sensitivity value up to 99.6% shows that it is difficult not to find alteration relations to MS in diseased individuals. The positive predictive value above 88.6% indicates the hit ratio of MS detection. And the negative predictive value above 86.3% shows the hit ratio of healthy people detection.

### 3.2. Performance Metrics of Classification Systems

It is relevant to mention that these results indicate that classifier systems differentiate the ECG signals of MS individuals from healthy people. Thus, ECG signal deviations found by classifiers indicate nonspecific changes of this syndrome. In this study, no specific analysis was conducted in depth on the contribution of each feature to changes in ECG signals. However, it was observed that the set of analyzed features may be altered due to the presence of MS.

However, according to Table 4, the training stage of the CNN was more time-consuming than the others classifiers—RobustBoost and SVM. It is important to mention that for the development of a real-time system, the important factor would be the testing time. The training time, which was actually short in this work compared to the convolutional network training times reported in the literature, does not affect the testing time.

In short, these results indicate that ECG signals can be used for effective and efficient MS syndrome detection. These individuals receiving an MS initial classification can then be referred to a health professional doctor to make clinical exams and investigate in more detail the MS factors, as well as for devising a proper treatment strategy. This follows the basic, standard idea of using computer classification tools as a dianostic aid and not as a final decision method [52].

Comparing these different classifiers, CNN clearly showed the best performance in terms of final objective metrics. One advancement of this technique with respect to the others is the automatic features extraction [53]. In addition, other classifiers used just some information extracted from peaks Q, R, and S, and other approaches for feature extraction can be tested. In fact, it is possible that other information, such as information related to QT and QTc intervals that are reported to change in diabetic individuals [54], as well as those related to P and T waves that are reportedly altered in the presence of MS [7], that may be important information for the development of these classifiers.

**Figure 4:**
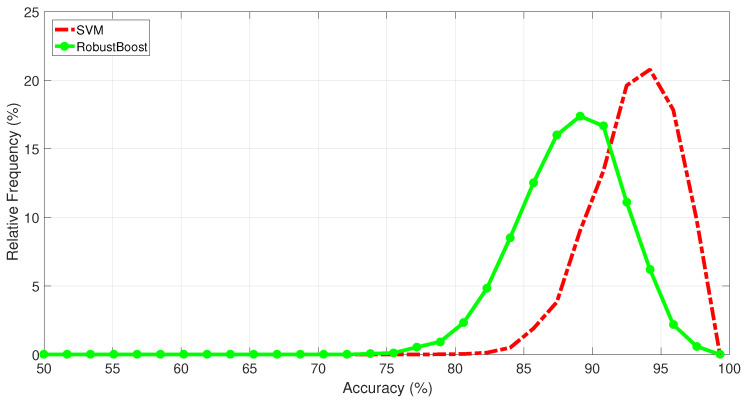
Relative frequencies of each observed accuracy value, both for SVMs and RobustBoost classifiers, in the ECG classification of basal stage. We used the *k*-fold cross-validation approach, with k=10, for 500 independent tests.

Also, Figure 4 shows the accuracy histogram of 500 training and testing tests, with k=10, using both SVM and RobustBoost classifiers. It is possible to see that the SVM had higher accuracy than RobustBoost on this research, with averages of about 94% and 89%, respectively.

For the classification of metabolic syndrome using ECG signals, it was demonstrated that even with limited data it is possible to achieve statistically significant results. Furthermore, it is important to mention that the robustness of the models was not formally tested under conditions with noise or artifacts in the ECG signals. However, these conditions were already present in the data used. Therefore, the robustness was indirectly tested.

### 3.3. Statistical Hypothesis Tests

According to the Kolmogorov–Smirnov test performed of the extracted ECG features, the data did not follow a Gaussian distribution, as we obtained p<0.001 in these tests. Therefore, we used a nonparametric statistical test for comparing features for the control and MS groups. Table 5 shows the *p* values we obtained regarding the null hypothesis that the features have the same median for both groups.

According to the current literature, cardiac alterations can happen in individuals with MS. Therefore, features extracted from ECG signals, such as information related to cardiac beats, to P waves, to QRS complexes, and to T waves can be correlated with MS presence [7,12,13]. In addition, individuals with MS can have left ventricular hypertrophy and commitment in diastolic function; these alterations can indicate an axis deviation in individuals with MS [55,56]. Therefore, the results of this work agree with the literature information.

The results summarized in Table 5 indicate that the features extracted from the DI and aVF leads were related to the mean and variances of divisions and intervals among Q, R, and S consecutive peaks and to the cardiac axis, thus rejecting the null hypothesis. This indicates that these features can differentiate individuals from the control group with respect to individuals of the metabolic syndrome group.

The association among analyzed ECG features and MS—as measured by Pearson correlations—varied among different time stages. The basal and 90-min stages showed the highest percentage of nonrelevant correlations. On the other hand, the 60-min stage showed the highest percentage of relevant correlations among ECG features and MS (Table 6).

Results from PCA showed that 13 eigenvectors represented most of the variability in the original dataset in every time stage available. After disregarding low-value coefficients in the eigenvectors, the minimum ECG features set with the best correlation scores was obtained in the 120-min stage—just 19 of the original 30 features, which were distributed as follows: the two of highest correlation scores; 9 out of 10 features with correlation in the intermediate range; and 8 out of 12 features with correlation values below 0.20 in the module, all with statistically significant correlations.

## 4. Conclusions

According to the scientific literature, the set of metabolic syndrome (MS) features is correlated with changes in the cardiovascular system [4,5,6,7,13,14]. However, we did not find many scientific articles mentioning automatic MS detection systems by means of electrocardiography signals exclusively. Because of this, this research attemped to fill this scientific gap.

Our investigation consisted of building different approaches to detect MS by means of ECG signals. These approaches included classifiers trained to indicate if a set of features of ECG signals is related to a control group or to individuals with MS. We started with a proposed set of algorithms to detect the QRS complex for feature extraction, using averages and variances of intervals and ratios between successive Q, R, and S peaks of the ECG signal. These features were used to train and test developed classifier systems, such as Support Vector Machines (SVMs) and RobustBoost classifiers. In addition, we also tested Convolution Neural Networks (CNNs), which constitute a state-of-the-art approach for raw signals and images classification [53].

Based on statistical analyses, the experimental results indicate that it is possible to detect MS from ECG signals using different approaches, with accuracies above 89.3%. Therefore, it is possible to use features related to Q, R, and S peaks to train SVM and RobustBoost classifiers. In addition, CNN models extract features automatically to classify MS with higher performance metrics compared to the other tested approaches. The methods tested in this study, particularly the CNN, are among the most recent techniques used in the state of the art.

Therefore, an electrocardiogram can be used to evaluate signals and indicate a possible diagnosis of MS. Then, the MS individuals may be referred to health professional for further evaluation and a detailed analysis of the MS factors by means of clinical exams.

In addition to these results, the research has some limitations. Individual anthropometric information regarding the participants of the database used in this research, such as glucose, triglyceride, cholesterol, blood pressure, weight, and age, was not found. Therefore, it was not possible to analyze the association of each MS risk factor with changes in the ECG. Thus, as a continuation of this research, in order to study the contributions of different MS components to the ECG, we intend to acquire new data from different individuals—with different combinations of MS components and including a control group of healthy people. In addition, we intend to add a group of individuals without MS but with a cardiovascular disease. The aim is to evaluate if these different groups of individuals may confuse the classifiers systems and change the statistical results. PCA and Person correlation score were able to individualize the performance of each ECG feature in the detection of MS in the machine learning approaches. On the other hand, exploring the validity of features in the performance of CNN is a field under development. This current work limitation will be assessed in future studies.

## Figures and Tables

**Table 1:** Risk factors of metabolic syndrome according to NCEP-ATP III criterion. Adapted from [3].

Risk Factors	Level
Waist circumference (men)	>102 cm
Waist circumference (woman)	>88 cm
Triglyceride	≥150 mg/dL
HDL Cholesterol (men)	<40 mg/dL
HDL Cholesterol (woman)	<50 mg/dL
Blood pressure	≥130 mmHg (systolic) or ≥85 mmHg (diastolic)
Fasting glycemia	≥110 mg/dL

**Table 2:** List and description of features extracted from the ECG signals using leads I and aVF.

	Features Extracted from the ECG Signals
1	mean of the RR intervals on the lead DI
2	mean of the RS intervals on the lead DI
3	mean of the QS intervals on the lead DI
4	mean of the QR intervals on the lead DI
5	mean of the cardiac axis, calculated based on the leads DI and aVF
6	mean of the division between R and Q on the lead DI
7	mean of the division between R and S on the lead DI
8	mean of the amplitude of R on the lead DI
9	variance of the RR intervals on the lead DI
10	variance of the RS intervals on the lead DI
11	variance of the QS intervals on the lead DI
12	variance of the QR intervals on the lead DI
13	variance of the cardiac axis, calculated based on the leads DI e aVF
14	variance of the division between R and Q on the lead DI
15	variance of the division between R and S on the lead DI
16	variance of the amplitude of R on the lead DI
17	mean of the RR intervals on the lead aVF
18	mean of the RS intervals on the lead aVF
19	mean of the QS intervals on the lead aVF
20	mean of the QR intervals on the lead aVF
21	mean of the division between R and Q on the lead aVF
22	mean of the division between R and S on the lead aVF
23	mean of the amplitude of R on the lead aVF
24	variance of the RR intervals on the lead aVF
25	variance of the RS intervals on the lead aVF
26	variance of the QS intervals on the lead aVF
27	variance of the QR intervals on the lead aVF
28	variance of the division between R and Q on the lead aVF
29	variance of the division between R and S on the lead aVF
30	variance of the amplitude of R on the lead aVF

**Table 3:** Means and standard deviations of performance metrics of the tested classifiers. We used the *k*-fold cross-validation method, with k=10. The classifiers include an SVM, a *RobustBoost* classifier, and a CNN. FPR means false positive rate (type I error), FNR is the false negative rate (type II error), PPV is the positive predictive value, NPV is the negative predictive value, Se is sensitivity, Acc is accuracy, and Sp is specificity.

	SVM	RobustBoost	CNN
FPR (%)	2.0% ± 2.9	11.4% ± 4.3	0.7% ± 0.6
FNR (%)	7.0% ± 4.8	5.5% ± 4.0	0.4% ± 0.6
PPV (%)	98.0% ± 2.9	88.6% ± 4.3	99.3% ± 0.6
NPV (%)	86.3% ± 9.4	90.4% ± 6.9	99.3% ± 1.0
Se (%)	93.0% ± 4.8	94.5% ± 4.0	99.6% ± 0.6
Acc (%)	93.8% ± 4.3	89.3% ± 3.5	99.3% ± 0.4
Sp (%)	95.8% ± 5.7	80.9% ± 6.0	98.9% ± 1.0

**Table 4:** Approximate time of algorithms execution using *k*-fold for cross-validation, with k=10. All tests used the same hardware and the same operating system. We intentionally used a comparatively simple, not state-of-the-art hardware and sofrware combination (I5 processor and 2GB RAM dedicated GPU).

Classifier	Time (Training)	Time (Testing)
SVM	0.3 s	0.02 s
RobustBoost	11 s	0.06 s
CNN	23 min	10 s

**Table 5:** Results, in terms of *p* values, of the result of the hypothesis test of each feature extracted from the ECG signals (shown in Table 2), related to control group and to group with MS, from different stages of OGTT.

	Basal	30 min	60 min	90 min	120 min
1	0.0	0.0	0.0	0.47	0.0
2	0.0	0.0	0.0	0.25	0.0
3	0.0	0.0	0.0	0.17	0.0
4	0.0	0.0	0.0	0.33	0.0
5	0.0	0.0	0.0	0.0	0.0
6	0.0	0.0	0.0	0.0	0.0
7	0.0	0.0	0.0	0.0	0.0
8	0.0	0.0	0.0	0.0	0.0
9	0.0	0.0	0.0	0.0	0.0
10	0.0	0.0	0.0	0.0	0.0
11	0.0	0.0	0.0	0.0	0.0
12	0.0	0.0	0.0	0.0	0.0
13	0.20	0.0	0.0	0.0	0.0
14	0.0	0.0	0.0	0.0	0.0
15	0.29	0.47	0.23	0.0	0.0
16	0.10	0.0	0.0	0.0	0.0
17	0.0	0.0	0.0	0.0	0.0
18	0.0	0.0	0.0	0.0	0.0
19	0.0	0.0	0.0	0.0	0.0
20	0.0	0.0	0.0	0.0	0.0
21	0.0	0.0	0.0	0.0	0.0
22	0.0	0.0	0.0	0.0	0.0
23	0.0	0.0	0.0	0.0	0.0
24	0.0	0.0	0.48	0.10	0.04
25	0.0	0.0	0.42	0.11	0.04
26	0.0	0.0	0.60	0.07	0.10
27	0.0	0.0	0.66	0.06	0.10
28	0.0	0.0	0.0	0.0	0.0
29	0.01	0.0	0.0	0.0	0.0
30	0.70	0.12	0.17	0.0	0.02

**Table 6:** Distribution of ECG features (described in Table 2) among ranges of Pearson correlation and *p* values in the group with MS from different stages of OGTT. Legend: n.a.—not available; NS—not significant.

*p* Values	Pearson Correlation Value in Module			% TOTAL FEATURES
	|r|<0.20	0.20≤|r|<0.50	0.50≤|r|<0.75	
p>0.05 (NS)	BASAL: 16; 22; 24; 25; 26; 27; 28; 29; 30	n.a.		BASAL: 30% (9/30)
	30 MIN: 6; 13; 16; 28; 29; 30			30 MIN: 20% (6/30)
	60 MIN: 6; 13; 14; 28; 30			60 MIN: 16.7% (5/30)
	90 MIN: 1; 2; 3; 4; 6; 16; 21; 28; 29			90 MIN: 30% (9/30)
	120 MIN: 15; 21; 28; 30			120 MIN: 13.3% (4/30)
0.001<p≤0.05	BASAL: 6; 21	n.a.		BASAL: 6.67% (2/30)
	30 MIN: 14; 21; 22			30 MIN: 10% (3/30)
	60 MIN: 15; 21; 29			60 MIN: 10% (3/30)
	90 MIN: 14; 15; 22; 30			90 MIN: 13.3% (4/30)
	120 MIN: 6; 9; 10; 11; 12; 13; 14; 24; 25; 26; 27; 29			120 MIN: 40% (12/30)
p≤0.001	BASAL: (2 of 30) 13; 15	BASAL: (15 of 30) 1; 2; 3; 4; 5; 7; 9; 10; 11; 12; 14; 17; 18; 19; 20	BASAL: (2 of 30) 8; 23	BASAL: 63.3% (19/30)
	30 MIN: (10 of 30) 7; 9; 10; 11; 12; 15; 24; 25; 26; 27	30 MIN: (9 of 30) 1; 2; 3; 4; 5; 17; 18; 19; 20	30 MIN: (2 of 30) 8; 23	30 MIN: 70% (21/30)
	60 MIN: (11 of 30) 7; 9; 10; 11; 12; 16; 22; 24; 25; 26; 27	60 MIN: (10 of 30) 1; 2; 3; 4; 5; 17; 18; 19; 20	60 MIN: (1 of 30) 8	60 MIN: 73.3% (22/30)
	90 MIN: (6 of 30) 7; 9; 10; 11; 12; 13	90 MIN: (9 of 30) 5; 17; 18; 19; 20; 24; 25; 26; 27	90 MIN: (2 of 30) 8; 23	90 MIN: 56.7% (17/30)
	120 MIN: (2 of 30) 16; 22	120 MIN: (10 of 30) 1; 2; 3; 4; 5; 7; 17; 18; 19; 20	120 MIN: (2 of 30) 8; 23	120 MIN: 46.7% (14/30)

## Data Availability

Data are contained within the article.
